# Aspects of brucellosis vaccination in Maranhão state

**DOI:** 10.3389/fmicb.2024.1504488

**Published:** 2024-11-27

**Authors:** Adriana Prazeres Paixão, Tânia Maria Duarte Silva, Sara Ione da Silva Alves, Carla Janaina Rebouças Marques do Rosário, Hamilton Pereira Santos, Danilo Cutrim Bezerra, Nancyleni Pinto Chaves Bezerra, Viviane Correa Silva Coimbra

**Affiliations:** ^1^Programa de Pós-Graduação Profissional em Defesa Sanitária Animal, Universidade Estadual do Maranhão, São Luís, Brazil; ^2^Departamento de Zootecnia, Universidade Estadual do Maranhão, São Luís, Brazil

**Keywords:** prevention, animal health, vaccination, public health, brucellosis

## Abstract

Bovine brucellosis is a zoonosis of great economic and public health relevance. Understanding the epidemiological aspects of this disease is a determining factor to help developing effective strategies and supporting decision-making processes. The aim of the present study is to carry out a situational vaccination diagnosis against brucellosis in Maranhão State’s cattle in order to identify challenges set for vaccination operations and to help the Official Veterinary Service to plan health interventions. In 2022, questionnaires focused on the socioeconomic profile and knowledge on vaccination were applied to the following groups to help achieving the aforementioned aims: (i) cattle breeders (*n* = 201), (ii) veterinarians registered with the National Program for Control and Eradication of Brucellosis and Animal Tuberculosis (*n* = 84), (iii) Official Veterinary Service servants involved in conducting the National Program for Control and Eradication of Brucellosis and Animal Tuberculosis (*n* = 75), and (iv) individuals in charge of vaccine retail outlets (*n* = 58). Farmers and those in charge of vaccine retail outlets presented average knowledge on this subject (51–70% accuracy). Yet, there were similar knowledge gaps in these two groups. Although the knowledge level recorded for registered veterinarians and Official Veterinary Service servants was high (71–100% accuracy), low adherence to personal protection equipment was also observed, and it is worrisome, given brucellosis’ zoonotic nature. In addition, veterinarians presented knowledge gaps when it comes to updates on the National Program for Control and Eradication of Brucellosis and Animal Tuberculosis Technical Regulations, and it can be an indicative that questionnaires were superficially or carelessly answered. Other obstacles to vaccination were poor quality of roads to access properties and breeders with limited number of bred calves in the age group 3 to 8 months. These barriers impair the expansion of vaccination cover against brucellosis in Maranhão State. The Official Veterinary Service must implement a strategic plan to fill out the knowledge gaps shown by those in this production chain and make it easier to get to the basis for effective health interventions aimed at brucellosis control in Maranhão State.

## 1 Introduction

Bovine brucellosis is a zoonosis of great economic and public health relevance featured by reproductive disorders such as abortions in the final three months of pregnancy. This disease has significant economic impact since it leads to reduced beef and milk production, longer time intervals between pregnancies and lower birth rates (Paulin, 2003). In total, 19,650 bovine brucellosis cases were reported in Brazil from 2014 to 2018, and 875 of them were recorded in Maranhão State (Brasil Ministério da Agricultura e Pecuária, 2020). Costs associated with treatment, animal loss, and need for disease control and prevention have considerable economic impact on farmers. Therefore, controlling and vaccinating against brucellosis is essential to mitigate the aforementioned issues and ensure animal production sustainability (Abebe et al., 2024).

Bacterium *Brucella abortus*, which is a Gram-negative and facultative intracellular coccobacillus, is the bovine brucellosis’ etiological agent. Its primary transmission takes place through horizontal routes, mucous membranes, and skin breaks. It can also happen through congenital vertical routes, although this rout is less common (Constable et al., 2016). Brucellas have affinity for erythritol, which is a sugar found in the placenta, mainly during the final three months of pregnancy. This sugar triggers an inflammatory process capable of both changing maternal-fetal junction necrosis and leading to abortion. In addition, other changes can be observed in infected animals, including joint injuries, orchitis and infertility (Riet-Correa et al., 2022).

Brucellosis poses risk not only to animal health, but to human health, as well. Approximately 2.1 million cases are reported on a yearly basis, worldwide, mainly in developing countries. Therefore, it is essential implementing more effective control and vaccination strategies to mitigate disease outspread (Laine et al., 2023). Brucellosis is similarly prevalent in neighboring countries like Argentina, and its rate ranges from 10 to 13% in bovine herds, and from4 to 5% infection in animals, individually. Annual economic losses due to this disease are estimated to reach $60 million due to abortions, reduced milk production and costs associated with disease control (Figueiredo et al., 2009).

The National Program for the Control and Eradication of Brucellosis and Animal Tuberculosis (PNCEBT) was launched by the Ministry of Agriculture and Livestock (MAPA) to reduce Brucellosis prevalence and incidence, as well as to make the Brazilian livestock competitive. Vaccination against brucellosis in calves in the age group 3 to 8 months, animal transportation and agglomeration control, positive animals’ culling and sanitation measures to prevent outbreaks are among the mandatory actions to be taken in Maranhão State (Brasil Ministério da Agricultura e Pecuária, 2017).

Vaccination is one of the main tools to control bovine brucellosis. Vaccines such as B19 and RB51 were effective in reducing abortions and disease incidence in herds. Attenuated vaccines used in brucellosis control programs are currently recommended by the World Organization for Animal Health (WOAH) (n.d.). Hikal et al. (2023) highlight that the main obstacle to eradicate brucellosis in domestic animals, in endemic and developing countries, is resistance to vaccination with living strains. Authors explore factors such as lack of sanitary control infrastructure, fear of vaccines’ side effects and producers’ distrust. These factors hinder adherence to vaccination programs and emphasize the importance of adopting an integrated approach and cooperation between public health and agricultural sectors.

Understanding the epidemiological aspects of this disease is a determining factor to help developing effective strategies and supporting decision-making processes. According to a seroepidemiological survey conducted in Maranhão State between 2007 and 2009, the prevalence of this disease reached 11.4% in herds and 2.5% in animals, individually (Borba et al., 2013). These rates classify the state as grade “D,” which corresponds to high-risk sanitary condition. Although actions to control and prevent brucellosis in Maranhão’s cattle herd have been taken, gaps remaining in this process still need to be addressed. The aim of the present study was to carry out a situational diagnosis of vaccination against brucellosis in cattle and buffaloes bred in Maranhão State in order to identify obstacles to vaccination operations and, consequently, to support health intervention planning by the Official Veterinary Service (OVS).

## 2 Materials and methods

### 2.1 Typology and study site

Observational descriptive qualitative-quantitative epidemiological study carried out based on collecting primary data through questionnaire application to target publics, namely: actors from the public and private system involved in PNCEBT.

**Figure 1:**
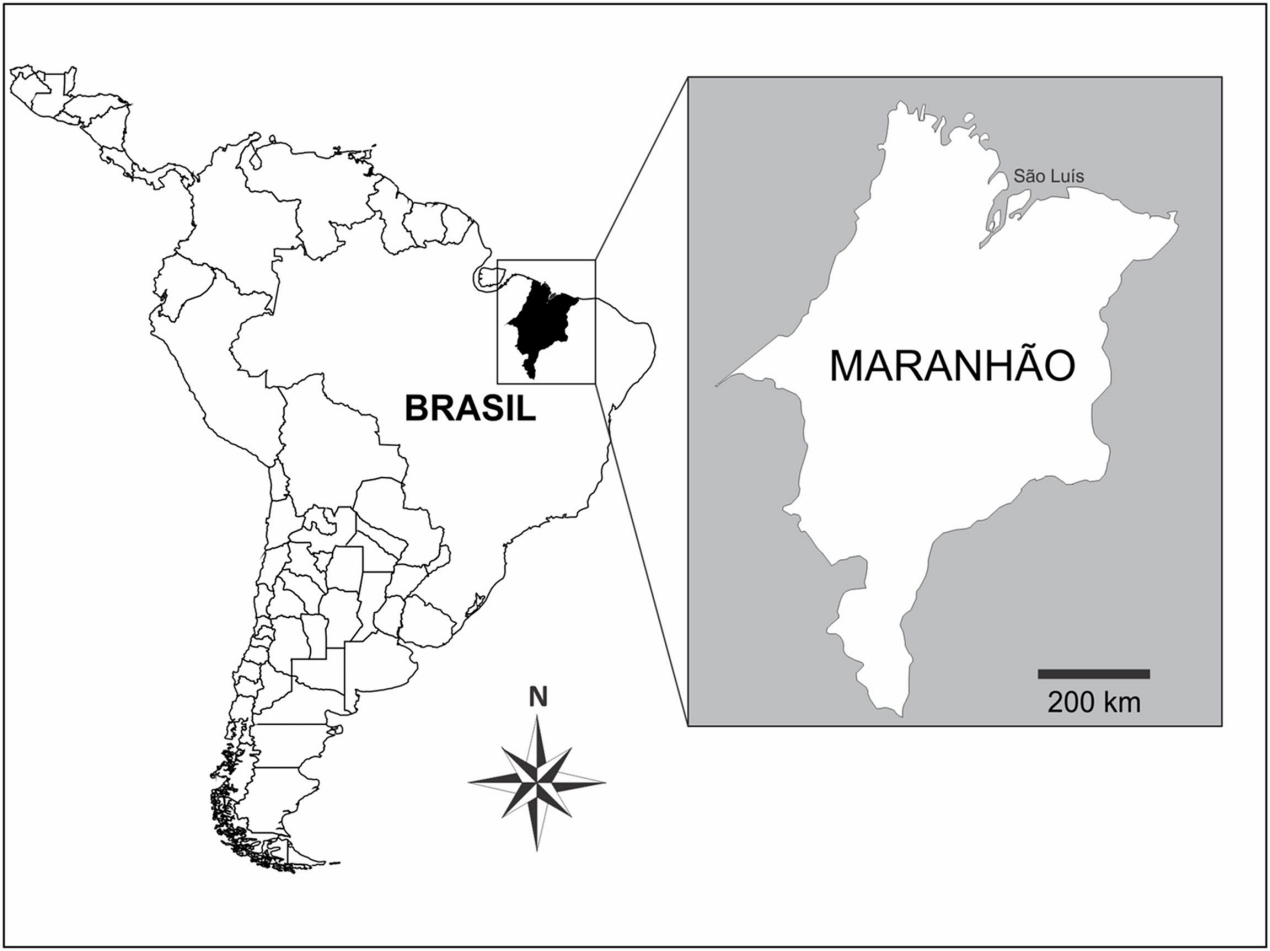
Maps showing Maranhão State, in Northeastern Brazil.

The study was conducted in Maranhão State, which is located 05°05′12” South and 42°48′42” West of Greenwich Meridian. Mean temperature in the state is close to 26°C and its mean rainfall reaches 197 mm (Atlas do Maranhão, 2006). Maranhão territory covers 331,936.949 km^2^ in Northeastern Brazil; it is limited by the Atlantic Ocean to the North; Tocantins State to the South and Southwest; Piauí State to the East, Northeast and Southeast; and Pará State to the West and Northwest. Its estimated population is 7,153,262 inhabitants, who are distributed in 217 municipalities that, in their turn, are grouped into five mesoregions: Northern, Western, Central, Eastern and Southern [Instituto Brasileiro de Geografia e Estatística (IBGE), 2023] (Figure 1).

### 2.2 Situational diagnosis of actors involved in the National Program for Control and Eradication of Brucellosis and Animal Tuberculosis

Specific questionnaires were developed for the situational diagnosis of bovine vaccination against brucellosis. They aimed at identifying the target public’s socioeconomic profile and knowledge level associated with vaccination against brucellosis, as well as at adopting health education practices and perceptions. Respondents were divided into four different groups: (i) breeders dealing with cattle farming, (ii) veterinarians registered with PNCEBT, (iii) OVS servants who work with PNCEBT conduction, and (iv) individuals in charge of brucellosis vaccine retail outlets. Questionnaires aiming at farmers were applied in person, and the others were applied in person and/or on the Google Forms^®^ platform, from January to December 2022.

According to Reports by Maranhão State’s OVS, the state has 143,670 cattle breeders, 339 veterinarians registered with the PNCEBT, 304 civil servants working in PNCEBT implementation and 131 brucellosis vaccine retail outlets [Agência Estadual de Defesa Agropecuária do Maranhão (AGED/MA), 2021]. The smallest sample size taken as representative of each target population was calculated by taking into consideration 10% margin error, at 95% confidence level. Therefore, 201 cattle breeders, 84 veterinarians registered with PNCEBT, 75 OVS servants and 58 individuals in charge of brucellosis vaccine retail outlets joined the research. All of them were selected by intentional non-probabilistic sampling, including respondents from the 18 managerial OVS Regional Units (RU).

The ratio of correct answers to questions in the questionnaires recorded for each assessed group was taken into account to qualify knowledge level regarding vaccination against brucellosis. They were separated into three ranges of correct answers: up to 50%—low knowledge, from 51 to 70%—average knowledge, and from 71 to 100% high knowledge.

### 2.3 Ethical aspects

The research complied with ethical precepts and followed all items in Resolution n. 196, from October 10, 1996, by the National Health Council. It was submitted to the Ethics Committee for Research with Human Beings (CEP/UEMA), through *Plataforma Brasil*, and approved through—Certificate of Presentation for Ethical Assessment (CAAE) n. 13753919.6.0000.5554. All participants were informed about the research aim and conditions. They signed the clarification and free consent form (TELC) before answering the questionnaire.

### 2.4 Data analysis

The gathered information was stored in Excel^®^ database, ordered and presented in Tables. Descriptive analyses were applied to all variables based on using ratio measurements. Graphics were plotted to help better visualizing some information.

## 3 Results and discussion

### 3.1 Situational diagnosis of cattle breeders

The socioeconomic features of cattle breeders joining the present research (*n* = 201) pointed towards the following profile: farm owners (69.6%), in the age group 41–60 years (51.8%), with complete high school (42. 8%), family income up to one minimum wage (47.8%), breeding 1 to 10 animals (40.5%), who have been cattle farming for more than 10 years (58.7%) and who are members of the Rural Workers Union (59.1%).

The observed profile highlighted small breeders, and this finding corroborates results in previous research, according to which, 85.14% rural properties in Maranhão State are featured by family farming (Silva et al., 2023). According to census data, 82.6% of farmers earn less than two monthly minimum wages; 12% of then earn 2.0 to 5.0 minimum wages and only 5% of these farmers manage to earn more than 5.0 minimum wages per month. The census also pointed out that more than 80% of agricultural properties are run by farmers belonging to the male sex, in the age group 40–45 years (26.3%). They are followed by those in the age groups 50–55 years (20.5%), 25–35 years (14.36%) and (11.22%) over 65 years [Instituto Brasileiro de Geografia e Estatística (IBGE), 2017].

Schooling was a positive element because 42.8% of assessed farmers had complete high school, and 8.9% of them had a college major. According to the latest agricultural census, 70% of the rural population did not finish elementary school and only 2% of it has a college major [Instituto Brasileiro de Geografia e Estatística (IBGE), 2017]. Studies have reported that people with higher schooling find it easier to embody new information and to accept new technologies to improve their activity (Cunha et al., 2012). It is also important knowing the prevailing age group and class representation to guide educational actions and to set the most appropriate methods.

**Figure 2:**
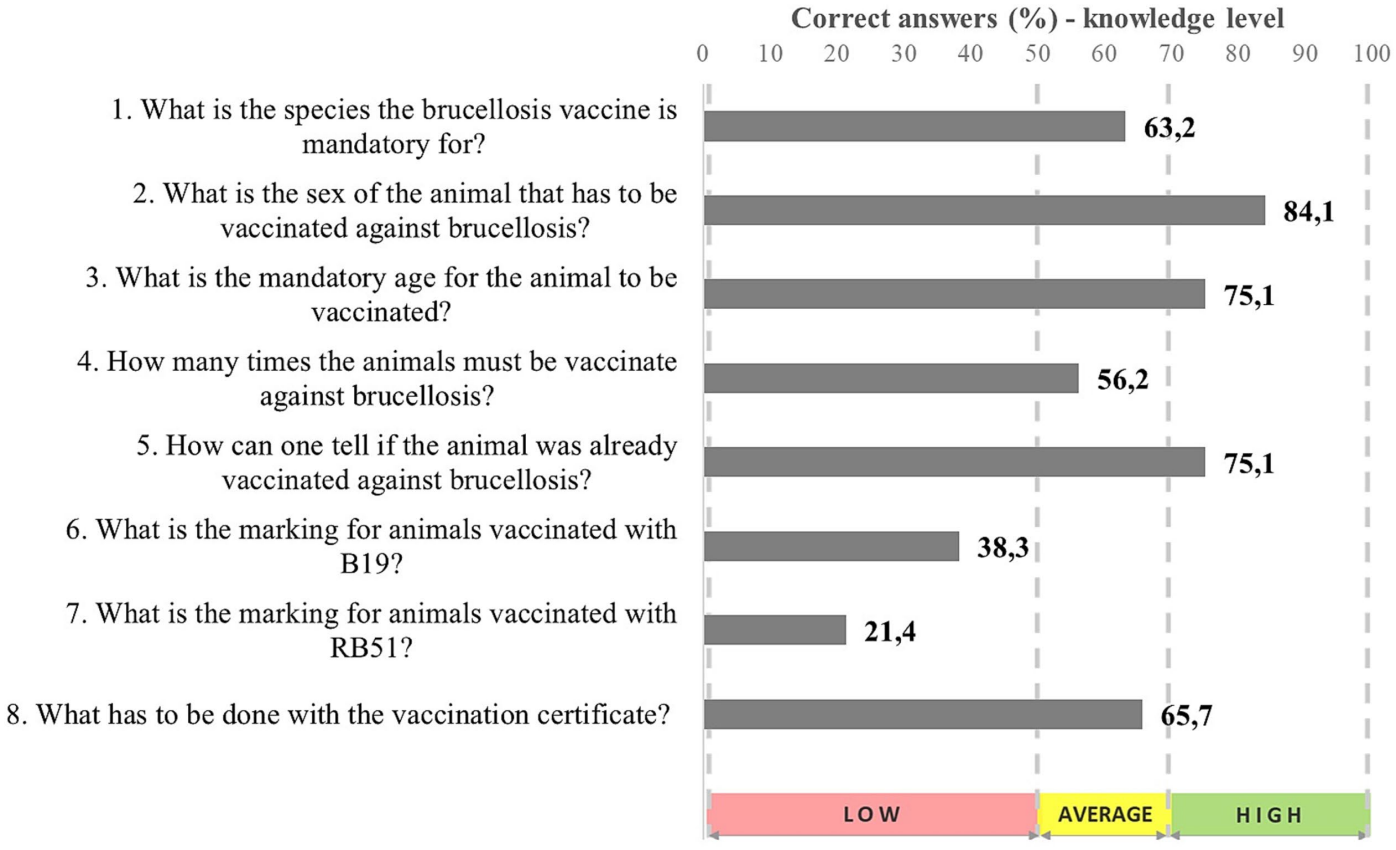
Knowledge level on vaccination against brucellosis recorded for cattle breeders in Maranhão State (*n* = 201), 2022.

In total, 146 (72.6%) of the 201 assessed breeders reported to vaccinate their animals against brucellosis as routine procedure. They were all assessed for the level of their knowledge on this practice. The assessments showed average knowledge on it, but their knowledge was low when more specific information was approached, such as animals’ marking after vaccination (Figure 2). Although animals’ post-vaccination hot branding is not a recommended practice by animal protection standards, besides requiring revisions of the Brazilian legislation, it remains a legal requirement. However, some states, such as São Paulo, have adopted one single branding process for animal-marking purposes.

According to Figure 2, most participants mentioned that female (84.1%) bovines (63.2%) should be vaccinated against brucellosis at the age of 3–8 months (75.1%), only once in life (56.2%). They acknowledged that the animal’s face is marked as vaccinated against brucellosis (75.1%). The last digit of the vaccination year must be marked on the left side of the animal’s face when it is vaccinated with the B19 dose (38.3%); a “V” must be marked on it when the animal is vaccinated with the RB51 strain (21.4%).

Most farmers are unaware of female buffalo’s mandatory vaccination against brucellosis. Only 32.3% of them knew that the vaccine is mandatory for this species. This is a quite worrisome finding if one bears in mind that Maranhão State houses the 4th largest buffalo herd in the country: approximately 89,000 animals [Instituto Brasileiro de Geografia e Estatística (IBGE), 2020]. Therefore, the target public must understand that representatives from this species must be vaccinated against brucellosis as mandatory procedure.

It was observed that 34.3% of assessed breeders reported that vaccinators account for collecting and transporting the waste generated by vaccination procedures. It is essential investigating the waste management practices adopted by these professionals to make sure of its appropriate final destination. Inadequate waste disposal in common trash cans or in municipal landfills can pose significant environmental and animal contamination risks, including the risk posed on humans. Therefore, it is necessary implementing strict waste management protocols to prevent its adverse impacts on both public health and the environment.

In total, 46.3% breeders were granted with the vaccination certificate right after the vaccination procedure. This certificate is used by most breeders (65.7%) to prove to OVS that this operation was done. It is essential mentioning that breeders have stated lack of knowledge about penalties (warnings and fines) provided on the current legislation they are subject to in case their cattle is not vaccinated against brucellosis (64.2%). Most participants would like to acquire more information on this subject (88.5%), although they stated to have already gotten guidance on the vaccination process by OVS servants (53.5%) or by independent veterinarians (15.4%). Most participants would like to get more information on this subject (88.5%).

Barbosa et al. (2016) assessed the level of breeders’ knowledge on brucellosis impact on public health, in Piauí State. They identified breeders’ lack of knowledge due to lack of awareness and clarification about this disease and damages caused by it.

### 3.2 Situational diagnosis of veterinarians registered with the official veterinary service

In total, 53.7% of the 84 veterinarians sampled in this study have official register to vaccinate against brucellosis, but only 46.4% of them are also qualified to diagnose this disease. Most veterinarians are in the age group 31–50 years (57.1%), work in providing assistance to rural properties (48.8%) or as technical managers (TM) in livestock (42.9%), and have family income ranging from 2 to 5 minimum wages.

**Figure 3:**
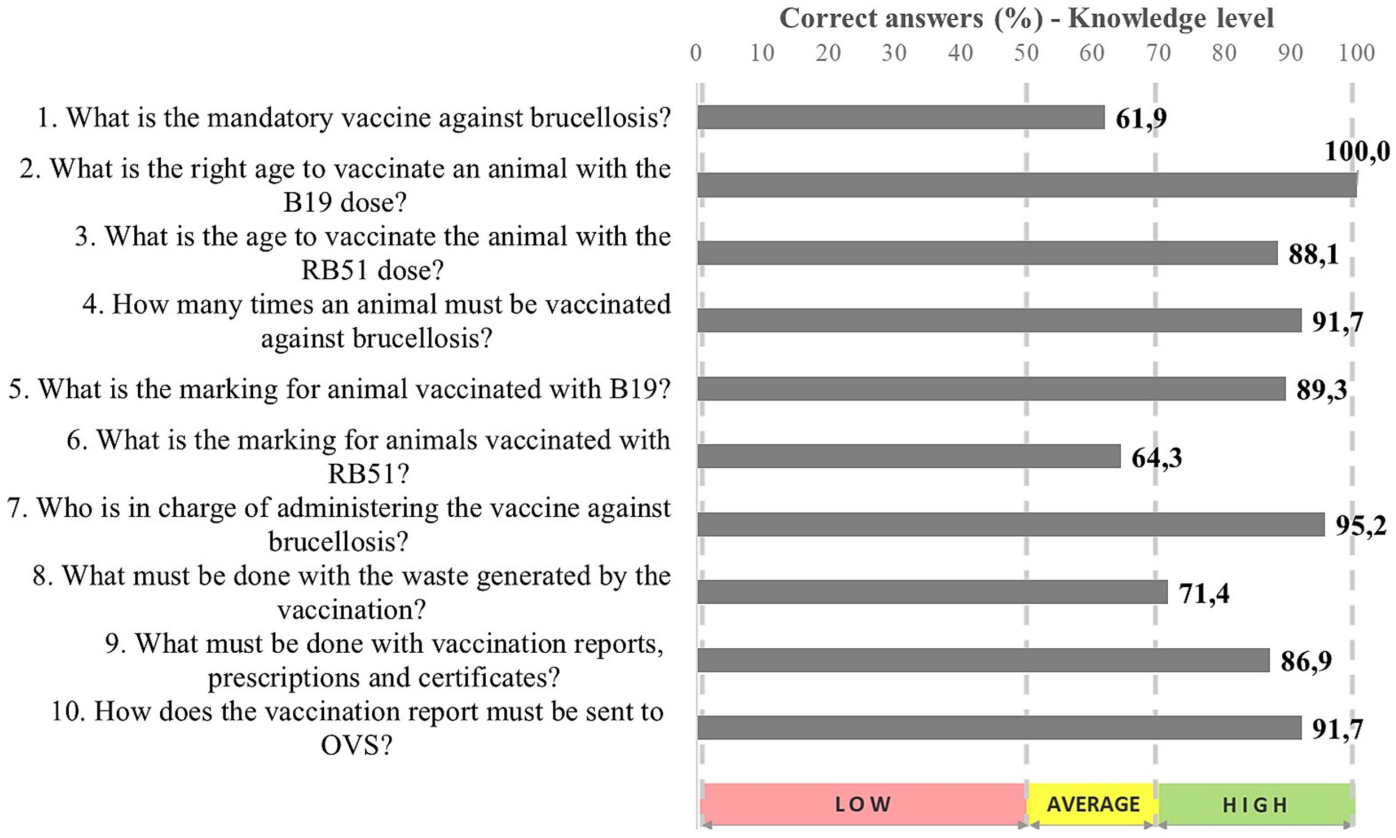
Knowledge level on vaccination against brucellosis recorded for veterinarians registered with the OVS in Maranhão State (*n* = 84), 2022.

Research points out that active veterinarians’ mean age is approximately 40 years old [Conselho Regional de Medicina Veterinária de São Paulo (CRMV/SP), 2021]. They are self-employed (30.2%) or work as private sector employees (23.3%) (Caldas, 2017) and earn up to five monthly minimum wages (67.1%) (Pereira et al., 2022). Figure 3 summarizes information on veterinarians’ knowledge about the vaccination practices against brucellosis carried out by them. It can be seen that the level of their knowledge on the most often addressed issues is high.

Clearly, 61.9% veterinarians are aware of the mandatory vaccination with the B19 strain in females in the age group 3–8 months (100%) (Figure 3). Normative Instruction n. 10/2017-MAPA recommends all bovine and buffalo females in the age group 3–8 months to be vaccinated with the B19 vaccine (single dose). In this case, the vaccine with the RB51 strain is optional for this age group and mandatory for bovine females over 8 months old, who were not previously vaccinated with B19 (Figure 3) (Brasil Ministério da Agricultura e Pecuária, 2017).

Most registered veterinarians (64.3%) understand that animals vaccinated with RB51 must be marked with a “V” on the left side of their faces, but a significant rate of them (28.6%) are not sure about the marking type to be adopted and 7.1% of them reported that animals should not be marked. This scenario may be associated with changes in PNCEBT Technical Regulations. From this time onwards, OVS has held technical meetings, lectures, *webinars*, interviews, live broadcastings, among others; however, according to reports by OVS servants, professionals’ participation in education events remains poor.

Participants’ perception about the need for using PPE showed that they use masks (79.8%), boots (38.1%), ropers (27.4%), apron or disposable coat over their clothes (14.3%) and transparent glasses (2.4%). Most vaccinators do not use some PPEs essential for vaccination against brucellosis; therefore, these agents are exposed to risk of contamination by vaccine and animal handling.

It is essential recalling that both vaccines (B19 and RB51) are made with living attenuated etiological agents (*Brucella*) that are pathogenic for humans. There are several reports in the literature about accidental infection with these agents, mainly among veterinarians. Therefore, it is crucial using basic PPEs, such as masks, protective glasses, gloves and long-sleeved apron, as well as disposable syringes and needles, during vaccination.

Nogueira et al. (2022) assessed the serum prevalence of anti-*Brucella abortus* antibodies in veterinarians in Mato Grosso State (MT) and associated risk factors. They found that most individuals among the reactive ones were independent veterinarians (40.3%) who had contact with the B19 and RB51 vaccines or who attended laboratories that diagnose brucellosis. According to Labarthe and Pereira (2008), general adherence to biosafety concepts will remain complicated as long as the veterinary community lacks deeper awareness of individual and collective protection knowledge associated with good practices.

When it comes to post-vaccination waste, 71.4% of participants stated to burn and bury the material used during vaccination processes in the property. The remaining ones stated to collect the waste and to take it with them for further disposal in the municipal landfill or in the property’s common trash. This finding corroborates observations reported by breeders and it deserves educational interventions that take into consideration environmental risks and the risk to public health posed by these practices.

Disposing veterinary-product bottles with common waste is an inappropriate practice given the occupational and environmental risk posed by these products’ features (Takayanagui, 2005; Güinther, 2010). According to the National Solid Waste Policy (Law 12,305/2010), all waste from human or animal healthcare-related services requires different management processes, in addition to environmentally appropriate final disposal (Brasil Ministério da Agricultura e Pecuária, 2018).

In some states, specific training for vaccination operations is not mandatory for veterinarians to register with the OVS. Only college degree and official registration with the professional council are required for it. It is important adopting prior and continuous training for these professionals given this activity’s specificities and the continuous updates in normative acts. It must be done to solve some doubts that had risen throughout the present study.

The assessed veterinarians answered that they are aware of the current status of the federal legislation about vaccination against brucellosis (95.2%). However, they stated to like to get updated information on this topic (96.4%). This finding was expected, if one bears in mind that professionals must idealize and seek dignifying conditions to exercise their profession, the best way possible, based on the legal parameters consistent with their Code of Ethics (Pazó and Heâncio, 2014).

The rural extension exercise is based on “diffusionism”, which mainly aims at outspreading new information and technological tools (Zuin, 2021). Thus, the relevance of independent veterinarians who also play educational role is highlighted, because these professionals’ participation in the process to build new reflections is a fundamental link, since it enables breeders to adopt good agricultural practices. This process explains the importance of being updated on animal health.

The following answers were highlighted when veterinarians were asked about difficulties faced in vaccination practices: distance from, and difficult access to, some properties (20.2%). As previously observed, small breeders prevail in Maranhão State. They often breed from 1 to 10 animals, and 1 or 2 of them are calves at the recommended age for vaccination against brucellosis. Therefore, registered veterinarians have high costs with the logistics to vaccinate few animals, and it is not compensating, mainly due to expenses with trips to rural properties.

Furthermore, the poor quality of roads to access small properties is an obstacle for small breeders, because most roads do not have asphalt paving and are prone to dust, vibrations and mud in the rainy season (Vieira, 2018).

### 3.3 Situational diagnosis of those in charge of brucellosis vaccine retail outlets

In total, 58 brucellosis vaccine retail outlets in Maranhão State were assessed and this procedure counted on massive participation of those responsible for vaccine retailing in Santa Inês and Itapecuru Mirim units. Most sampled individuals owned their outlets (41.2%), but the questionnaire was answered by these establishments’ managers or technical managers (43.5 and 15.3%, respectively), in some cases.

A study aimed at featuring agricultural retailing in the main Brazilian states and assessed 123 vaccine retail outlets through a questionnaire applied to those in charge of these establishments. According to the answers, these outlets adopt similar operations, regardless of the region they are located in. They have broader reach in states accounting for a smaller number of municipalities, such as Maranhão State. These retail outlets only count on their owners and on some employees (*n* = 45; 37%); moreover, the owners manage the vaccination operations, themselves (Negri, 2022).

This survey also identified that these retail outlets have been around for 20 years, on average, and most of them have been operating for 11–30 years. Retail outlets were classified as micro-enterprises (*n* = 63; 51%), with up to 9 employees; or as small companies (*n* = 51; 42%), with 10 to 49 employees (Negri, 2022).

Respondents’ socioeconomic features in the present study led to the following profile: age group from 31 to 40 years (52.3%), college major (36.8%), family income ranging from 2 to 5 minimum wages, being in the market for approximately 10 years (44.6%) and the establishment as the main income source (77%).

Brucellosis vaccine is sold all year long (65.4%), at most retailers. Respondents’ reported to inform OVS (52.4%) about the arrival of new doses when they were asked about procedures to be adopted for vaccine acquisition. Therefore, they can release the immunogens for proper packaging and organization in the establishment’s cold chain. Yet, they carry out daily stock control in spreadsheets (32.8%) or in the establishment’s own network system (22.5%) to monitor dose inflow and outflow.

OVS control over retail outlets includes vaccine income, purchase invoice checking, temperature and storage control, and prescription filing. Stock control and sales management of immunobiological products are carried out through the state’s Agricultural Management System (SIGAMA).

According to current regulations, retail outlets must be registered with both OVS and the Federal Superintendence of Agriculture and Livestock (SFA/MAPA) to sell B19 and RB51 vaccines. This register is subject to prescription issuance by a veterinarian registered with the PNCEBT. This register must be available for 1 year at the commercial establishment for OVS inspection purposes (Brasil Ministério da Agricultura e Pecuária, 2017).

The assessed retail outlets only sell vaccines upon the presentation of the prescription issued by a veterinarian registered with OVS (68.2%). These prescriptions are filed in suspended folders (57.3%) or in other organization procedures (29.7%) adopted by establishment managers. Vaccines are mainly sold in 15-dose bottles (46.6%) delivered to buyers in ice Styrofoam boxes (71.4%).

The B19 vaccine is available in vials containing 10, 15 and 50 doses, in lyophilized form, accompanied by the diluent. RB51 is available in vials of 15 and 25 doses, also containing the diluent (Paulin, 2003). Most buyers demand 15-dose vials, as already highlighted, mainly due to the large number of small properties holding small numbers of animals in the region.

Similar survey was carried out in Iporá municipality (GO) and it showed that 77.8% (n = 100) agricultural stores mainly target small and medium-sized breeders, and 22.2% of them assist all breeders, including the large ones (Feitosa Júnior et al., 2022). Retail outlets are often breeders’ first contact when a disease case is diagnosed in their herd. It is so, because retail outlets are popular places to purchase agricultural inputs, as well as to seek solutions for plantation, installation and herd issues (Negri, 2022; Moura et al., 2014).

**Figure 4:**
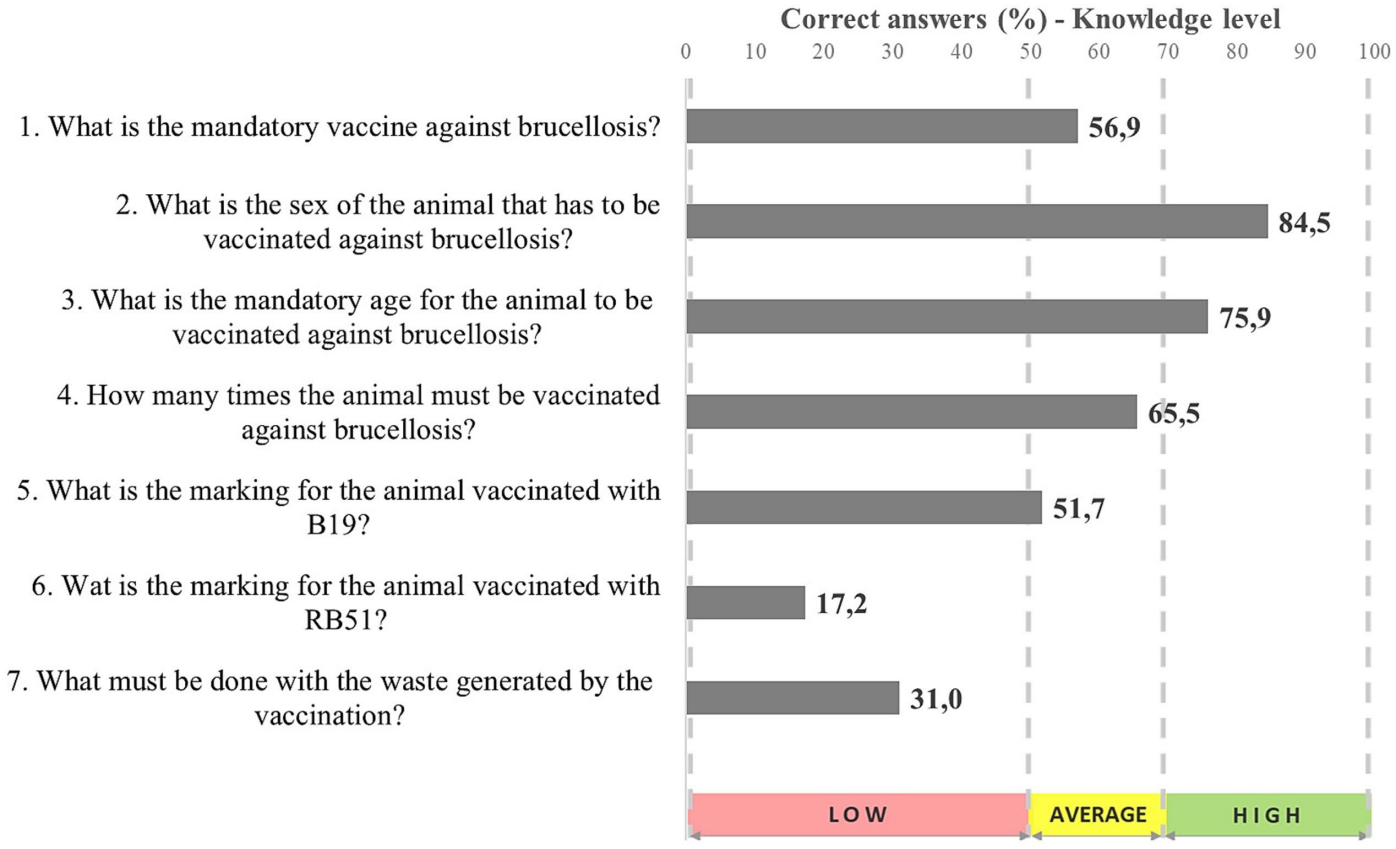
Knowledge level on vaccination practices against brucellosis recorded for those responsible for vaccine retail outlets in Maranhão State (*n* = 58), 2022.

Thus, those in charge of these establishments, and their employees, must be updated on several topics related to animal health, since they work as multiplier health-information agents. Accordingly, individuals sampled in the current study were assessed for their level of knowledge on vaccination against brucellosis (Figure 4).

According to the respondents, B19 vaccination is mandatory against brucellosis (56.9%) in females (84.5%) in the age group 3 to 8 months (75.9%) who must be vaccinated only once in life (65.5%). Over half of them know that females vaccinated with B19 must have the last digit of the vaccination year marked on the left side of their faces (51.7%), but only 17.2% of them were able to inform the marking to be adopted for animals vaccinated with RB51. Only 31% of respondents were aware of the appropriate disposal for the waste generated during vaccination procedures (Gaspar et al., 2015).

Similar to participants in the present study, those in charge of retailing vaccines have average knowledge about basic information on his topic and low knowledge when it comes to more specific subjects, such as the marking type adopted for vaccinated animals and appropriate vaccination waste disposal.

Respondents stated that they had already received orientations on vaccination against brucellosis by OVS servants (34.6%) or by retailers’ technical managers (30.8%). They added that they would like to get more information on this topic (90.4%).

Accordingly, educational diagnoses can work as tool to help spreading teachings and public knowledge, deficiencies and difficulties, in order to reestablish actions seeking positive outcomes (Cony Filho et al., 2016). Health education is an instrument to promote health; it uses different methodologies to develop and exchange knowledge, and to integrate agricultural services to all parts involved in this chain [Instituto de Defesa Agropecuária e Florestal do Espírito Santo (IDAF), 2022; Valente et al., 2009]. OVS accounts for promoting continuous education as first step towards inspection services.

### 3.4 Situational diagnosis of official veterinary service servants working at the National Program for Control and Eradication of Brucellosis and Animal Tuberculosis

In total, 41 (54.7%) of the 75 OVS servants working at PNCEBT were assessed: 41 (54.7%) state agricultural inspectors (veterinarians), 19 (25.3%) inspection technicians, 4 (5.3%) inspection assistant and 11 (14.7%) outsourced employees. Most respondents had a major degree (75.1%) and family income higher than 5 minimum wages (49.2%).

The aliquot of 7.5% sampled servants are in charge of vaccination against brucellosis in municipalities facing shortage of veterinarians registered with the state. This activity is provided by OVS in areas lacking registered independent veterinarians or in regions that do not fully meet the Program’s demands (Brasil Ministério da Agricultura e Pecuária, 2017).

**Figure 5:**
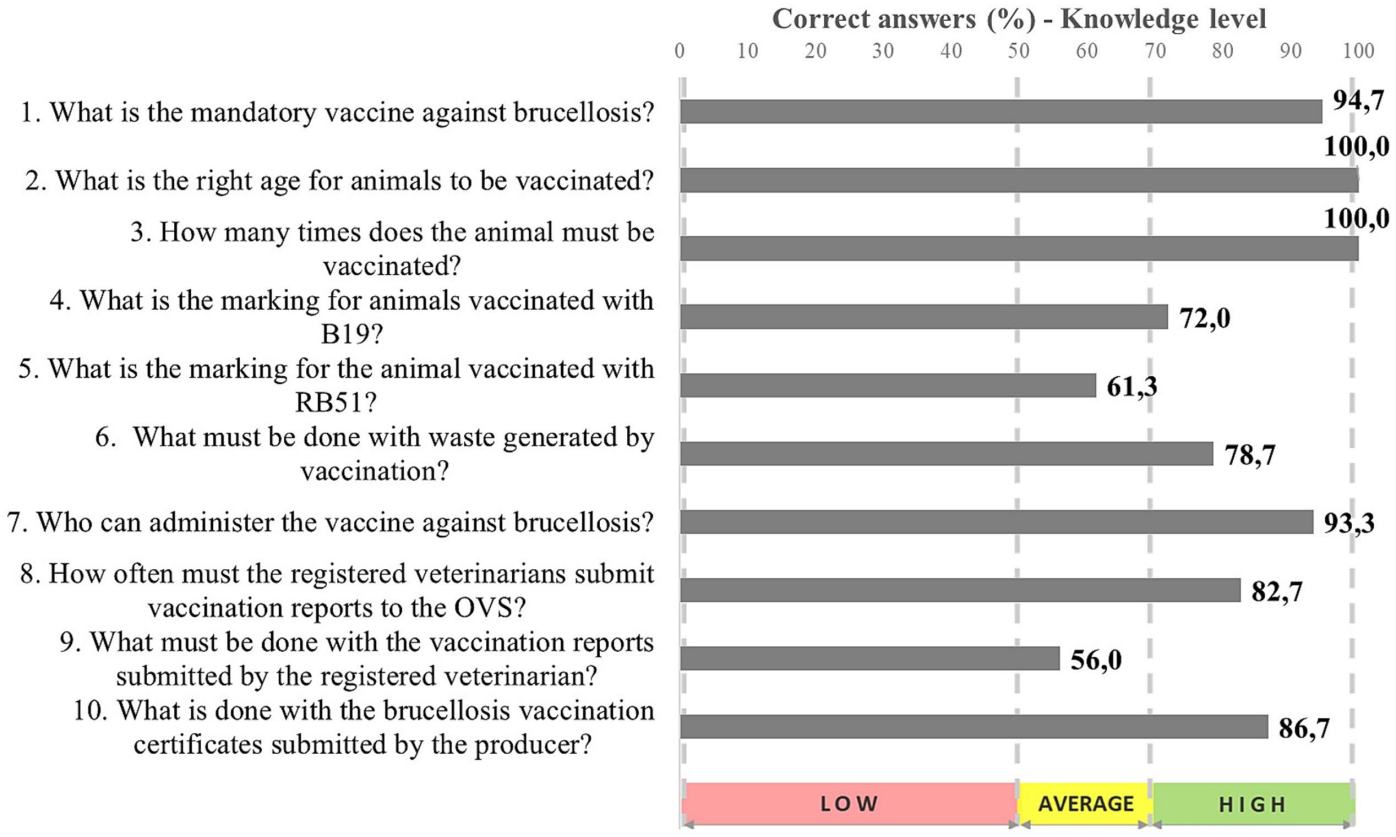
Knowledge level on vaccination against brucellosis recorded for official veterinary service (OVS) servants in Maranhão State (*n* = 75) 2022.

Servants were assessed for their knowledge on vaccination against brucellosis. There was high knowledge on most addressed items (Figure 5). Almost all respondents (94.7%) reported that the B19 vaccine is mandatory for all females in the age group 3–8 months (100%), and that it must be administered only once in life (100%). The sampled ones knew that RB51 is mandatory for females over 8 months old who have not been vaccinated with B19. Most of them pointed out that marking must be carried out on the animal’s face after vaccination with the B19 (72%) and RB51 (61.3%) doses. They also knew that veterinarians must make sure that the waste generated during vaccination is properly disposed of (78.7%).

Servants were asked about PPE, which must be used when carrying out vaccination protocols, and the following answers were recorded: gloves (91.2%), pants and short-sleeved shirt (84.5%), boots (71.3%), rompers (55.4%), transparent protective glasses (36.7%) and mask (3.2%). Servants are unaware of some essential PPEs to protect the health of professionals involved in vaccination against brucellosis, such as masks, protective glasses and long-sleeved aprons. Continuous training on the fundamentals and mechanisms of the toxic action of several chemical substances related to these professionals’ practices, in association with good biosafety practices, must be provided.

This group acknowledges that only registered veterinarians, and their assistants, can carry out the vaccination procedure (93.3%), and that they must submit vaccination activity reports to local OVS offices, on a monthly basis (82.7%). According to them, reports are filed in the office and a copy of them is sent to both the Regional Unit (UR) and the central office (56%). Yet, they reported that certificates were used by breeders to prove vaccination, and these reports are filed with the registry (86.7%), for audit purposes. Servants’ high knowledge level was clear; however, investment in continuous educational projects remains essential.

Researchers mentioned that servants working in agricultural defense programs need training to qualify for analyzing, understanding and for having a critical and ethical vision about health actions to be developed, in addition to argue about the relevance of their role in society (Bezerra et al., 2021).

**Figure 6:**
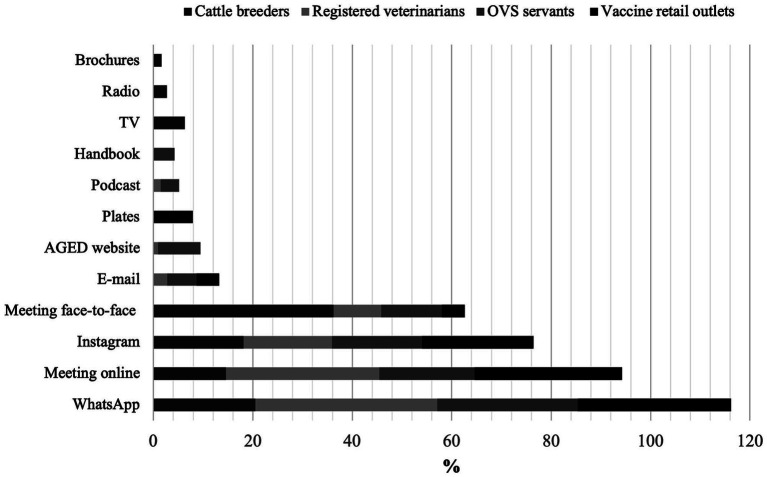
Preference on how to get technical information about vaccination against brucellosis recorded for cattle breeders, registered veterinarians, those responsible for vaccine retail outlets and OVS servants (*n* = 418) in Maranhão State, 2022.

Finally, all four herein assessed groups were asked about their preference for getting information about vaccination against brucellosis (Figure 6). Most of them preferred social networks, such as WhatsApp^®^ and Instagram^®^, which allow quick, effective and direct reach to those interested in passing on health information. This item was followed by synchronous interactions through face-to-face or online meetings.

In 2017, a survey carried out by the Brazilian Association of Rural Marketing and Agribusiness (ABMRA), which holds 2,835 breeders from fifteen Federative Units (UF), showed that 61% of all respondents had a cell phone with access to the internet and that WhatsApp^®^ was the most used social media; it reached 96% of those who have access to the internet. In 2020, 94% of this public had a smartphone, and it proves the expansion of digital media for its direct access [Zuin, 2021; Associação Brasileira de Marketing Rural e Agronegócio (ABMBRA), 2017].

Knowing the preferences of the target public about access to information is important at the time to plan the best ways and means to reach it and to promote its interest in the addressed topics. Accordingly, using participatory techniques and dialogic communication is an essential tool for the educational process, in addition to allow OVS to use this information in decision-making processes regarding educational actions in agricultural defense (Feitosa Júnior et al., 2022; Brasil Ministério da Agricultura e Pecuária, 2022).

## 4 Conclusion

According to the results, cattle breeders in Maranhão State presented average knowledge about vaccinating calves against brucellosis, and this finding shows lack of necessary information about procedures to be taken before, during and after vaccination. Lack of knowledge and/or doubts were similar among those in charge of selling vaccines, who also presented average knowledge about it. This outcome highlights this group’s need for updates, since they are in constant contact with breeders and are information replicators.

Although the level of knowledge of registered veterinarians and OVS servants about vaccination against brucellosis was considered high, there was low adherence to PPE using. This is a worrisome fact, if one bears in mind that brucellosis is a zoonotic disease. It is worth mentioning that registered veterinarians showed lack of knowledge about recent changes in legislation; therefore, it is recommended to adopt updating training for veterinarians and assistants registered with PNCEBT and a mandatory course for professionals interested in joining the Program—the course should be provided by AGED/MA or by similar bodies. The poor quality of roads to access small properties was also highlighted as obstacle to broader vaccination coverage.

WhatsApp is recommended to be used as strategic tool to share reliable and accessible information. Creating specific content on safe vaccination practices and legislative updates based on support provided by experts and communication professionals can make the understanding and engagement of various stakeholders in the production chain easier. Organizing this information into short and direct messages spread on WhatsApp can significantly improve adherence to the best vaccination practices. This approach can also help overcoming logistical and access challenges faced by the most isolated rural properties, as well as contributing to strengthening health interventions aimed at controlling brucellosis in Maranhão State.

## Data Availability

The original contributions presented in the study are included in the article/supplementary material, further inquiries can be directed to the corresponding author.
